# Ten-Eleven Translocation (TET) Enzymes Modulate the Activation of Dendritic Cells in Allergic Rhinitis

**DOI:** 10.3389/fimmu.2019.02271

**Published:** 2019-09-26

**Authors:** Hang Li, Tong Lu, Wei Sun, Renqiang Ma, Hua Zhong, Yi Wei, Dehua Chen, Yihui Wen, Chris Carlsten, Weiping Wen

**Affiliations:** ^1^Department of Otorhinolaryngology-Head and Neck Surgery, The First Affiliated Hospital of Sun Yat-sen University, Guangzhou, China; ^2^Guangzhou Key Laboratory of Otorhinolaryngology-Head and Neck Surgery, The First Affiliated Hospital of Sun Yat-sen University, Guangzhou, China; ^3^Air Pollution Exposure Laboratory, Division of Respiratory Medicine, Department of Medicine, University of British Columbia, Vancouver, BC, Canada

**Keywords:** allergic rhinitis, dendritic cell, DNA hydroxymethylation, epigenetics, ten-eleven translocation

## Abstract

**Background:** The prevalence of allergic rhinitis (AR) has increased in recent decades. Accumulating evidence indicates that aberrant DNA demethylation modulated by enzymes of ten-eleven translocation (TET) promotes an imbalanced immune response.

**Objective:** This study aimed to explore TETs on the activation of dendritic cells (DCs) in AR.

**Methods:** The levels of TETs in peripheral blood mononuclear cells (PBMCs), peripheral myeloid DCs (mDCs), and plasmacytoid DCs (pDCs) from house dust mite (HDM)-sensitive AR patients and healthy volunteers (HC) were evaluated by qPCR and flow cytometry. The levels of 5-hydroxymethylcytosine (5hmC) and 5-methylcytosine (5mC) in PBMCs were determined by DNA-5hmC and DNA-5mC ELISA. The major HDM allergen, Dermatophagoides pteronyssinus (Der p 1), was used to stimulate atopic monocyte-derived DCs (moDCs) to assess its effect on the TETs. TET1 knockdown effect on the activation of non-atopic and atopic moDCs was investigated.

**Results:** TETs and global 5hmC were higher in PBMCs of AR than HC. So was TET1 in peripheral mDCs and pDCs of AR. *In vitro*, TET1 in atopic moDCs was significantly decreased by allergen challenge. Knockdown of TET1 in moDCs tended to induce CD86, CD80, and CD40 in AR but not in HC. TET1-knockdown moDCs significantly decreased the differentiation of activated regulatory T cells in AR.

**Conclusion:** DCs from AR patients express higher TET1 and are susceptible to be activated by TET1 decrease, which can be triggered by allergen challenge. Collectively, this suggests a role for TET in the pathogenesis of AR and potential for novel TET1-related, preventive, and therapeutic targets.

**Graphical Abstract:**
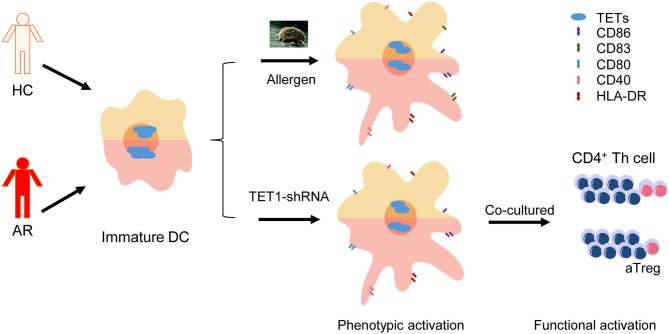
Dendritic cells (DCs) from allergic rhinitis (AR) patients express higher Ten-eleven translocation Methylcytosine Dioxygenase 1 (TET1) and are susceptible to be activated by TET1 decrease, which can be triggered by allergen challenge.

## Introduction

The prevalence of allergic rhinitis (AR), which has become a global public health problem, is ~10–40%, and the annual cost of AR treatment in the United States alone is about $5 billion (1–3). AR is an IgE-mediated nasal inflammation driven by type 2 helper T (Th2) cells, resulting from allergen exposure in a sensitized individual (4, 5).

Dendritic cells (DCs) are critical antigen-presenting cells and known as the coordinators between innate and adaptive immune (6, 7). Growing studies demonstrate that blood DCs are immature and can be recruited to airway mediating allergic airway inflammation (8, 9). Blood DCs have been defined as myeloid dendritic cells (mDCs, including more common mDC-1 and extremely rare mDC-2) and plasmacytoid dendritic cells (pDCs) (10). After allergen challenge, immature DCs can increase costimulatory molecules and secrete a series of cytokines to directly activate T-helper cells and indirectly inhibit the immunosuppressive regulatory T cells (7, 11, 12). Dermatophagoides pteronyssinus (Der p 1), the major antigen of the common allergen house dust mite (HDM), was found to increase costimulatory molecule CD86 on atopic DCs, but not non-atopic ones, inferring the dysregulation of DC might be responsible for the development of allergic diseases (13).

With the increasing morbidity of AR in the past few decades, epigenetics, which constitutes a bridge between genetic and environmental factors, has been proposed to regulate the pathophysiology and pathogenesis of AR (14, 15). Importantly, DNA methylation, the first defined and the most well-known epigenetic mechanism (16), has been suggested to take part in the development of AR (14, 17–19). For example, Nestor et al. (18) showed that the DNA methylation pattern in CD4^+^ T cells separated seasonal AR patients from healthy donors. After allergen challenge, hypermethylation of SLFN12 gene in human peripheral blood mononuclear cells (PBMCs) was induced and correlated with increased clinical rhinitis symptoms (14). Moreover, Li et al. (19) demonstrated that hypomethylation of IL13 gene was associated with higher risk of HDM-sensitized AR. These studies suggest that aberrant DNA methylation could be a biomarker and a potential therapeutic target of AR.

Ten-eleven translocation (TET) enzymes, including TET1, TET2, and TET3, are a family of dioxygenases (20). They have been demonstrated to convert 5-methylcytosine (5mC) to 5-hydroxymethylcytosine (5hmC) and mediate DNA demethylation during mammalian development and disease pathogenesis, including inflammation, myeloid malignancies, and tumors (21–24). Recently, studies have shown that TET-mediated DNA demethylation plays a vital role in the differentiation of monocytes (25–27). However, the role of TET-mediated DNA demethylation in the differentiation and function of DCs and their roles in AR are unclear.

In this study, we wonder if the levels of TETs in the peripheral DCs from AR patients and healthy volunteers are different. Secondly, whether TET enzymes are involved in mediating Der p 1 challenge effect in monocyte-derived DCs (moDCs). Moreover, knockdown of TET1, which is predominant among TETs in DCs, was conducted to investigate its role in the phenotypic and functional activation of moDCs, and whether it's allergic status specific.

## Methods

### Subjects

Ninety-four diagnosed HDM-sensitive AR patients and 38 healthy volunteers were recruited in this study. The clinical characteristics of the participants were shown in Table 1. The summary of the sample quantity used in each experiment was listed in Table S1. The diagnosis of HDM-sensitive AR was based on the Allergic Rhinitis and Its Impact on Asthma (ARIA) guidelines, including clinical symptoms of sneezing, watery rhinorrhea, congestion, and mono-sensitization to Der p 1, as determined by specific IgE (sIgE, HOB Biotech Group, Suzhou, China). The sIgE of all healthy volunteers were under 0.35 kU/L. Exclusion criteria for AR and healthy volunteers were as follows: (1) using anti-histamine drugs or glucocorticoids within 2 weeks preceding sampling, (2) with a history of specific allergen immunotherapy, (3) with respiratory infection or chronic rhinosinusitis, (4) with immunodeficiency or other diseases related to immune abnormality, (5) pregnancy. Specimens and clinical data were collected with informed consent under protocols approved by the Ethics Committee of the First Affiliated Hospital of Sun Yat-sen University, Guangzhou, China (Approval No. 2016197).

**Table 1:** Clinical characteristics of participants in the observational study.

	**Healthy volunteers** ** (*n* = 38)**	**Allergic rhinitis** ** (*n* = 94)**	***p* value**
Age (year), mean ± SD	29.27 ± 5.09	27.35 ± 6.76	>0.05
Sex, male/female	18/20	45/49	>0.05
Total IgE concentration (kU/L), mean ± SD	58.86 ± 39.87	100.97 ± 58.44	<0.01
Der p sIgE concentration (kU/L), mean ± SD	0.10 ± 0.07	49.61 ± 70.18	<0.0001

### Quantitative Real-Time PCR (qPCR)

The mRNA expression levels of TET1, TET2, and TET3 in PBMCs were evaluated using qPCR. PBMCs were isolated, from peripheral blood of the subjects, by Ficoll-Hypaque density gradient centrifugation with Lymphocyte Separation Medium (MP Biomedicals, Santa Ana, USA). The detailed description is presented in the Supplementary Materials.

### Quantification of 5mC and 5hmC

Genomic DNA was isolated from PBMCs by using E.Z.N.A. Genomic DNA isolation kit (Omega Bio-Tek). Global 5mC and 5hmC content in the PBMCs were quantified with 5mC DNA ELISA Kit and Quest 5hmC DNA ELISA kit (ZYMO, USA). The detailed information is presented in the Supplementary Materials.

### Generation of moDCs

PBMCs were isolated from 6 non-atopic healthy volunteers and 6 HDM-sensitive AR patients, as mentioned above. The clinical characteristics of the participants were shown in Table 2. CD14^+^ cells and CD4^+^ cells were isolated from PBMCs using CD14 Microbeads and CD4 Microbeads (Miltenyi Biotec, Bergisch Gladbach Germany). The cells were seeded into a 48-well plate at a density of 2.5 × 10^5^ cells/well. Complete medium, which was made up of RPMI-1640 (Gibco), penicillin/streptomycin/L-glutamine (Gibco) and 10% fetal bovine serum (FBS, Gibco, US origin), was used to culture CD4^+^ T cells for 7 days and the medium was changed every 2 days. To induce DCs from CD14^+^ cells monocytes, complete medium supplemented with recombinant human granulocyte-macrophage-colony-stimulating factor (GM-CSF, 50 ng/mL; R&D Systems, Minneapolis, USA), and interleukin 4 (IL-4, 10 ng/mL; R&D Systems) was used. On day 5, the immature moDCs were stimulated by 1 μg/mL Der p 1 for 48 h. All experiments were conducted in a humidified 5% CO_2_ atmosphere at 37°C.

**Table 2:** Clinical characteristics of participants included in the *in vitro* study.

	**Healthy volunteers** ** (*n* = 6)**	**Allergic rhinitis** ** (*n* = 6)**	***p* value**
Age (year), mean ± SD	22.67 ± 1.97	22.00 ± 2.45	>0.05
Sex, male/female	3/3	3/3	>0.05
Total IgE concentration (kU/L), mean ± SD	48.68 ± 31.10	258.70 ± 146.30	<0.01
Der p sIgE concentration (kU/L), mean ± SD	0.06 ± 0.08	101.10 ± 67.45	<0.01

### Adenovirus-Mediated Gene Transfer

In order to knock down TET1 expression in moDCs, TET1 short hairpin RNA (shRNA) was packed in recombinant adenovirus GV119 vector constructed by GeneChem (Shanghai, China). TET1-shRNA sequences were as follows:

hTET1[shRNA#1]: CTTTGCTAGTGCAGTGTAT,
hTET1[shRNA#2]: ACACAACTTGCTTCGATAATT,
hTET1[shRNA#3]: CCTATATGTATGGCACAATAT.

For infection, moDCs were induced according to the protocols noted above, but in 96-well round bottom plate (2 × 10^4^ cells/well). On day 5, immature moDCs were transfected with adenovirus at 300 multiplicity of infection (MOI) in the presence of polybrene (5 μL/mL, Cyagen biosciences, Guangzhou, China). After another 48 h of culture, moDCs were collected for flow cytometric determination or further studies.

### Autologous Mixed Lymphocyte Reactions (AMLR)

The ability of TET1-inhibited moDCs to induce the proliferation of CD4^+^ T cells was evaluated using AMLR. MoDCs that had been transfected for 48 h were counted and co-cultured with autologous CD4^+^ T cells at a ratio of 1:10 (1 × 10^4^: 1 × 10^5^) in 96-well round bottom plates. Cells were co-cultured in RPMI-1640 (Gibco), penicillin/streptomycin/L-glutamine (Gibco) and 10% fetal bovine serum (FBS, Gibco, US origin) at 37°C in 5% CO_2_ for another 3 days. The proliferation of CD4^+^ T cells was assessed by flow cytometry with carboxyfluorescein diacetate succinimidyl ester (CFSE) (eBioscience, San Diego, CA, USA) labeling.

### Flow Cytometry and Antibodies

**Figure 1:**
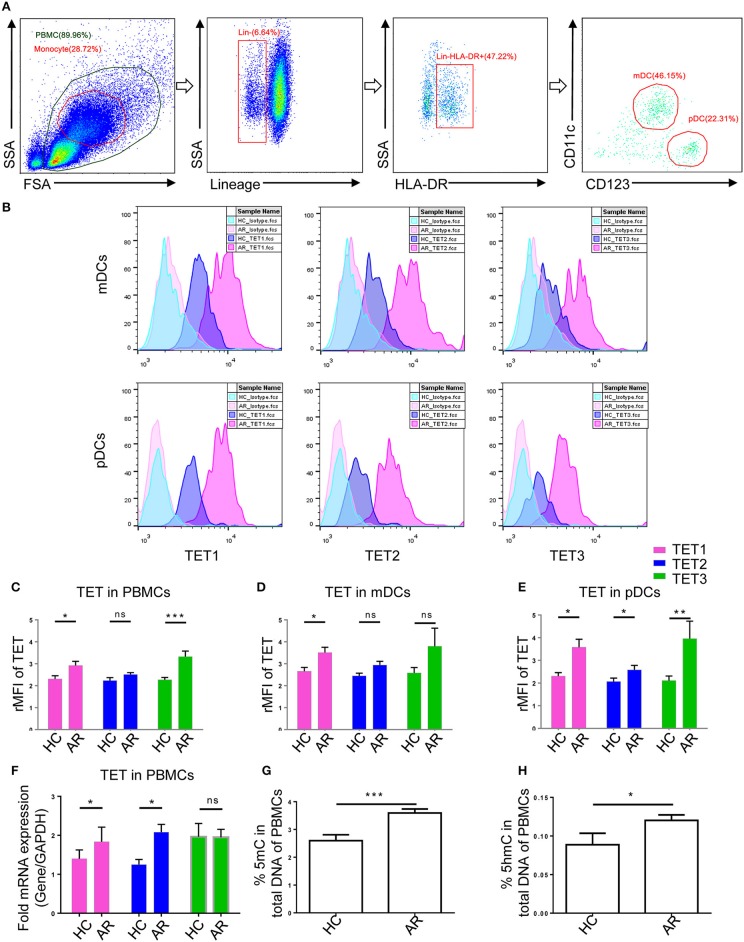
Ten-Eleven Translocation (TET1, TET2, and TET3) family enzymes are associated with allergic rhinitis (AR) in human patients. **(A)** Representative gating strategies of flow cytometry analyses for peripheral blood mononuclear cells (PBMCs) and Lin^−^HLA-DR^+^CD11c^+^CD123^−^ myeloid DCs (mDCs) and Lin^−^HLA-DR^+^CD11c^−^ CD123^+^ plasmacytoid DCs (pDCs). **(B)** Representative histogram plots of TET1, TET2, and TET3 expression in mDCs and pDCs. **(C–E)** Expression of TET enzymes in PBMCs **(C)**, mDCs **(D)**, and pDCs **(E)** in healthy controls (HC) and AR patients. The relative median fluorescence intensity (rMFI) was defined as the median fluorescence intensity (MFI) of TET divided by the MFI of Isotype. **(F)** TET family mRNA expression levels in PBMCs of healthy controls and AR patients. **(G,H)** Global 5mC **(G)** and 5hmC **(H)** levels in PBMCs of healthy controls and AR patients. Values were presented as means ± SEMs. Mann-Whitney tests were conducted. ^*^p < 0.05, ^**^ *p* < 0.01, and ^***^ *p* < 0.001.

To determine TET enzymes expression in human peripheral DCs, the following anti-human antibodies were used: anti-Lineage Cocktail FITC (CD3/14/16/19/20/56) (Biolegend), anti-HLA-DR Brilliant Violet 421™ (Biolegend), anti-CD11c PE (Biolegend), anti- CD123 PE/Cy7 (Biolegend), anti-TET1 (1:1000, ab191698, Abcam), anti-TET2 (1:1000, ab94580, Abcam), anti-TET3 (1:1000, ab139311, Abcam), and Brilliant Violet 510™ Donkey anti-rabbit IgG (Biolegend). PBMCs were collected as mentioned above and incubated with surface marker antibodies at 4°C for 30 min. According to the manufacturer's instructions of Foxp3/Transcription Factor Staining Buffer Set (Thermofisher Scientific), cells were fixed at RT for 30 min and washed twice with 1X permeabilization buffer. The cells were incubated with intracellular primary antibodies (TET1, TET2, or TET3, 1:250) at RT for 30 min. After washing with 1X permeabilization buffer, the cells were incubated with Brilliant Violet 510™ Donkey anti-rabbit IgG antibody (1:300) at RT for 30 min. Cells were washed and examined by flow cytometry. Gating strategies are shown in Figure 1A. Representative histogram plots with the isotype control are shown in Figure 1B. The relative median fluorescence intensity (rMFI) was defined as the median fluorescence intensity (MFI) of TET divided by the MFI of Isotype control.

To determine TET enzymes expression after allergen challenge *in vitro*, the following anti-human antibodies were used: anti-CD1a PE-Cyanine7 (eBioscience), anti-TET1, anti-TET2, anti-TET3, and Brilliant Violet 510™ Donkey anti-rabbit IgG. Staining process was followed, as mentioned previously. TET enzymes expression were analyzed in CD1a^+^ cells.

To evaluate the phenotypic activation of moDCs, moDCs on day 7 were harvested and stained with the following anti-human antibodies: anti-CD1a PE-Cyanine7, anti-CD83 FITC, anti-CD80 (B7-1) PE-Cy5, anti-CD86 (B7-2) PE, and anti-HLA-DR-eFluro®450 (All from eBioscience). Cells were collected and incubated with surface marker antibodies at 4°C for 30 min.

To explore the effect of TET1-knockdown moDCs on the differentiation of regulatory T cells (Tregs), transfected-moDCs were co-cultured with autologous CD4^+^ T cells at a ratio of 1:10 (1 × 10^4^: 1 × 10^5^) in 96-well round bottom plates at 37°C in 5% CO_2_ on day 7 for another 72 h. Anti-CD4 PerCP-Cy5.5, anti-CD45RA eFluor450, and anti-FoxP3 PE antibodies were used. Staining process was referred to the instruction manual of Foxp3/Transcription Factor Staining Buffer Set (Thermofisher Scientific). Samples were analyzed using a flow cytometer (CytoFLEX S by Beckman Coulter, California, USA) and the CytExpert Software (Beckman Coulter).

### Western Blot Analysis

Western blot analysis was performed to analyze the protein levels of TET1, TET2, and TET3 Cells in PBMCs and moDCs, and verify the transfection efficiency of TET1-shRNA. TET1 (1:1,000, ab191698, Abcam, Cambridge, MA, USA), TET2 (1:1,000, ab94580, Abcam), TET3 (1:1000, ab139311, Abcam), and HRP-conjugated GAPDH monoclonal antibody (1:10,000, proteintech) were used in this experiment. The detailed description is presented in the Supplementary Materials.

### Statistical Analysis

Statistical analyses were carried out with GraphPad Prism 6 software (La Jolla, California, USA). Data are presented as means ± SEM in the histogram. To compare TET enzymes expression between healthy volunteers and AR patients, the Mann-Whitney tests were conducted. Paired *t*-tests were used to analyze the two groups of paired data. Friedman test and Dunn's multiple comparisons tests were used to analyze more than two groups of paired data. A *p* < 0.05 was considered to indicate a significant difference.

## Results

### Expression of TET Family in PBMCs and DCs of AR Patients

In order to explore TET family expression levels in PBMCs of AR patients, we isolated PBMCs from the AR patients and healthy volunteers and analyzed the levels of TET1, TET2, and TET3 with flow cytometry and qPCR. Protein levels of TET1 and TET3 were higher (*p* = 0.04 and *p* = 0.0004, respectively) in the PBMCs of AR patients than that of healthy volunteers; TET2, although appearing higher in AR patients, was not significantly different between two groups (*p* = 0.07) (Figure 1C). As DCs play an important role as a bridge in innate immunity and adaptive immunity, we evaluated TET expression specifically in DCs and found TET1 was significantly higher in both peripheral mDCs and pDCs (*p* = 0.03 and *p* = 0.01), while TET2 and TET3 were higher in pDCs (*p* = 0.04 and *p* = 0.001) of AR patients than healthy controls. Levels of TET2 and TET3 in mDCs of AR patients also appeared to be higher than that of healthy volunteers, but were not significantly so (*p* = 0.1 and *p* = 0.1) (Figures 1D,E). The mRNA expression of TETs was verified by qPCR. The mRNA levels of TET1 and TET2 were significantly higher in the PBMCs of AR patients (*p* = 0.03 and *p* = 0.01) compared with those of healthy volunteers (Figure 1F). These results indicated that TET family, especially TET1, may participate in the pathogenesis of AR.

### Global 5mC and 5hmC Levels in the PBMCs of AR Patients

TET enzymes catalyze the conversion of 5mC to 5hmC (20, 28). We next determined 5mC and 5hmC content in PBMCs of AR patients and healthy volunteers using 5mC and 5hmC DNA ELISA. Global 5mC and 5hmC levels were significantly higher in the PBMCs of AR patients compared with those of healthy volunteers (*p* = 0.0003 and *p* = 0.01) (Figures 1G,H). These results further suggest a role of DNA methylation and hydroxymethylation in AR.

### Allergen Stimulation Downregulated TET1 Expression in Atopic moDCs

**Figure 2:**
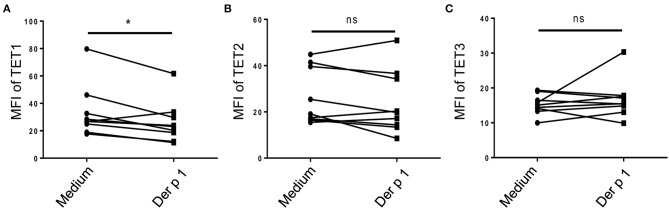
Allergen stimulation decreased TET1 in atopic monocyte-derived DCs from AR patients. Immature moDCs (day 5) induced from the peripheral blood monocytes of house dust mite (HDM)-allergic AR patients were exposed to Der p1 for 48 h and the expression of TET family (**A** showed TET1, **B** showed TET2, and **C** showed TET3) were assessed by flow cytometry. Paired *t*-tests were conducted to analyze the data. ^*^p < 0.05 (*n* = 8).

To explore whether TET family enzymes are involved in the activation of atopic moDCs, we isolated monocytes from PBMCs of HDM-sensitive AR patients and induced them into moDCs. After challenged by allergen for 48 h, TET1 expression in atopic moDCs was significantly decreased by Der p 1 (*p* = 0.02, Figure 2A), but TET2 and TET3 expression showed no obvious changes after allergen challenge (*p* = 0.2 and *p* = 0.4, Figures 2B,C). Our finding implies the specific effect of allergen challenge on TET1 expression in atopic moDCs.

### TET Family Expression in Human PBMCs and DCs

**Figure 3:**
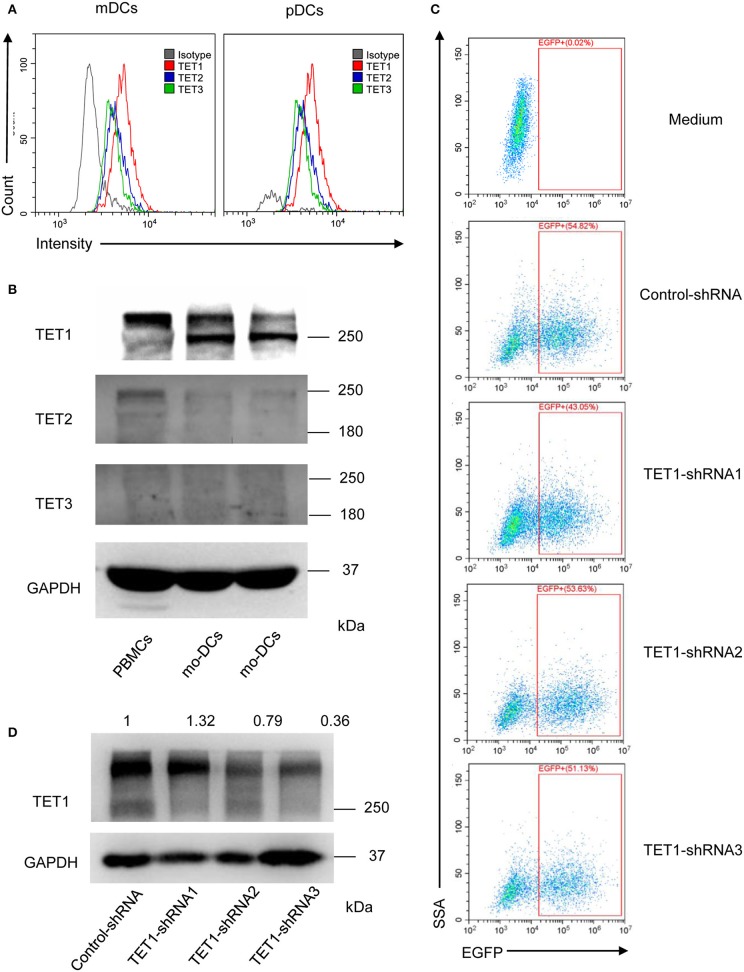
The relative expression of TET1, TET2, and TET3 in PBMCs and DCs and selectively knocked down the expression of TET1 with transfection of recombinant adenovirus. **(A)** The histogram shows TET1 expressed highest among TET family in human peripheral mDCs, and pDCs by flow cytometric analysis. **(B)** Comparing to TET2 and TET3, TET1 expressed highest in both PBMCs and moDCs. **(C)** Infection efficiency of recombinant adenovirus in moDCs. **(D)** Expression of TET1 in adenovirus-transfected moDCs was assessed using western blot. GAPDH was used as an internal control.

To further investigate the role of TET proteins in human PBMCs, we compared the relative expression of TET1, TET2, and TET3 with flow cytometry (Figure 3A) and Western Blot (Figure 3B). Ji et al. showed that the mRNA expression of TET1 was low and without detectable changes during the induction of moDCs (29). However, our results showed in the protein level, TET1 expression is predominant among TET family members in peripheral DC subsets and moDCs. Base on these intriguing results, we further knocked down TET1 in moDCs to investigate its role in the maturity and function of DC. As shown in Figures 3C,D, 50–60% of moDCs were transfected by TET1-shRNA3, and the expression of TET1 was successfully inhibited (~ 60% inhibition) after 48 h of transfection.

### TET1 Knockdown Effect on the Activation of moDCs in AR and HC Groups

**Figure 4:**
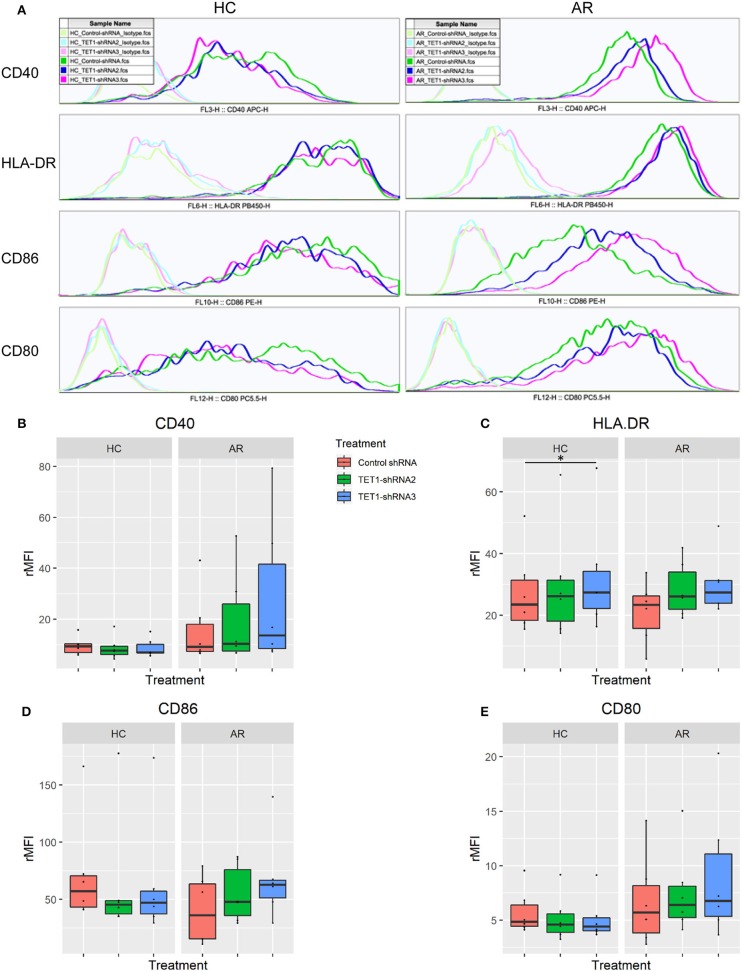
TET1 knockdown effect on phenotypic activation of non-atopic and atopic moDCs. Immature non-atopic and atopic moDCs (day 5) were transfected by control-shRNA, TET1-shRNA2, or TET1-shRNA3 adenovirus. After another 48 h, the expression of costimulatory molecules, including CD 40, HLA-DR, CD86, and CD80, were evaluated by flow cytometry. **(A)** The histograms show the typical comparisons of every costimulatory molecule expression among Control-shRNA-treated moDCs (dark green), TET1-shRNA2 (dark blue), and TET1-shRNA3-treated moDCs (magenta) and their isotype controls (light green, light blue, and pink, respectively). **(B–E)** Statistical analyses were conducted with Friedman test and Dunn's multiple comparisons tests (*n* = 6 HC and 6 AR) ^*^p < 0.05.

**Figure 5:**
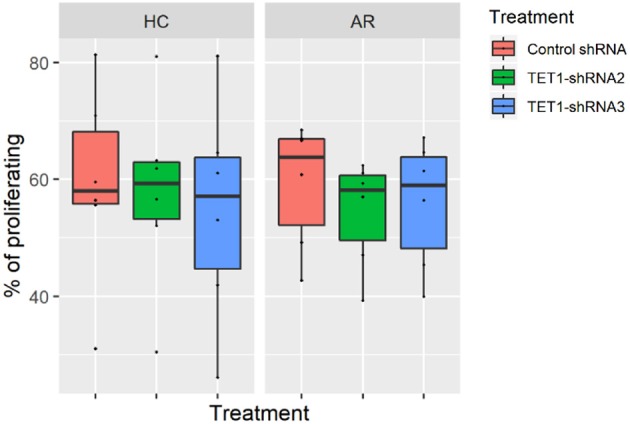
Effect of TET1-inhibited moDCs on the proliferation of CFSE-labeled T helper (Th) cells. Immature non-atopic and atopic moDCs (day 5) were transfected by control-shRNA, TET1-shRNA2, or TET1-shRNA3 adenovirus. On day 7, different treatments of moDCs were counted and co-cultured with carboxyfluorescein diacetate succinimidyl ester (CFSE)-labeled autologous Th cells at a ratio of 1:10 (1 × 10^4^: 1 × 10^5^) for another 72 h. The proliferation of CD4^+^ T cells measured by CFSE dilution. Statistical analyses were conducted with Friedman test and Dunn's multiple comparisons tests (*n* = 6 HC and 6 AR).

To evaluate the role of TET1 in the phenotypic activation of moDCs, costimulatory molecules expression were determined by flow cytometry after 48 h of transfection. Representative histograms are shown in Figure 4A. CD40, CD86, and CD80 appear to be increased by TET1 inhibition in AR (*p* > 0.05), but no such trends in HC (Figures 4B–E). HLA-DR was significantly induced in TET1-shRNA3 treatment in HC (*p* < 0.05). After another 3 days of AMLR, CFSE-labeled CD4^+^ T cells were collected for analysis. The proliferation of CD4^+^ T cells show no significant changes by TET1 inhibition, neither in HC nor AR groups (Figure 5).

### TET1-Inhibited moDCs Suppress the Activated Regulatory T Cells in AR

**Figure 6:**
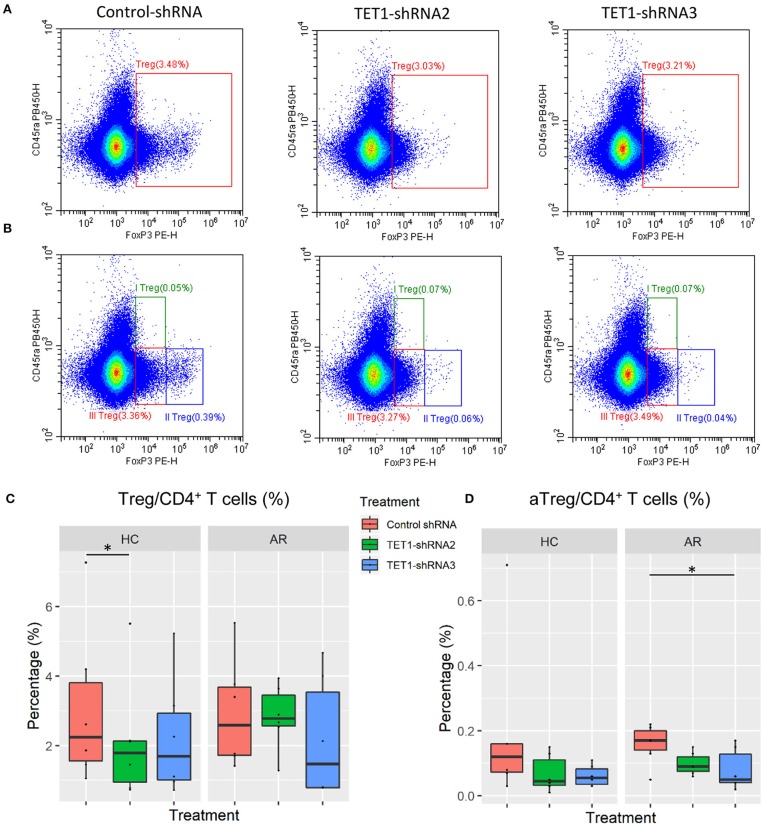
Effect of TET1-inhibited moDCs on the differentiation of regulatory T cells. Immature non-atopic and atopic moDCs (day 5) were transfected by control-shRNA, TET1-shRNA2, or TET1-shRNA3 adenovirus. On day 7, moDCs were co-cultured with autologous CD4^+^ T cells at a ratio of 1:10 (1 × 10^4^: 1 × 10^5^) for another 72 h. The proportion of CD4^+^FoxP3^+^ Tregs in CD4^+^ T cells **(A)**, and the proportion of Treg subsets, including CD4^+^CD45RA^+^FoxP3^lo^ resting Treg cells (rTregs, shown as I Treg), CD4^+^CD45RA^−^FoxP3^high^ activated Treg cells (aTregs, shown as II Treg) and CD4^+^CD45RA^−^FoxP3^lo^ non-suppressive T cells (shown as III Treg), in CD4^+^ T cells **(B)** were evaluated. **(C,D)** Statistical analyses were conducted with Friedman test and Dunn's multiple comparisons tests (*n* = 6 HC and 6 AR) ^*^p < 0.05.

Sakaguchi and his colleagues delineated regulatory T cells (Tregs) into three functionally distinct subsets, including CD45RA^+^FoxP3^lo^ resting Treg cells (rTregs), CD45RA^−^FoxP3^hi^ activated Treg cells (aTregs), and cytokine-secreting CD45RA^−^FoxP3^lo^ non-suppressive T cells and proved that aTregs were immune suppressive *in vitro* (30). We co-cultured the transfected moDCs with naïve CD4^+^ T cells to evaluate the effect of TET1-inhibited moDCs on the differentiation of Tregs and Treg subsets (Figures 6A,B). Surprisingly, TET1-inhibited moDCs significantly decreased the percentage of aTregs in AR groups, which might be linked to the immune activation in AR (Control-shRNA vs. TET1-shRNA3, *p* < 0.05, Figure 6D). And no significant effect was found on the proportion of rTregs and non-suppressive T cells in CD4^+^ T cells (shown in Figure S2). Meanwhile, TET1-inhibited moDCs decreased Tregs in HC group (Control-shRNA vs. TET1-shRNA2, *p* < 0.05, Figure 6C). These results suggest that TET1 is associated with the immune regulation of DC in crosstalk with other immune cells, such as the immunosuppressive aTregs in AR.

## Discussion

It has been postulated that not only the changes in the genetic background but also the influence of environmental factors contributed to the increased prevalence of allergic diseases over the past several decades (31). Key environmental risk factors, such as diesel exhaust particles, O_3_, SO_2_, and NO, have been widely proved to be associated with allergic disease exacerbation and development (32–35). We have demonstrated that enhancer of zeste homolog 2 (EZH2), which is known as a histone-lysine N-methyltransferase enzyme, its expression in circulating DCs was negatively correlated with the treatment time of allergen immunotherapy in AR patients (36). It is increasingly clear that gene-environment interaction plays a pivotal role in the susceptibility, pathogenesis, and therapy of AR (37, 38). However, the role of TET enzymes-mediated DNA demethylation in the process of the immune response in AR has not been fully elucidated.

The finding of higher TET1, TET3, and 5hmC in PBMCs of AR patients suggested that TET family and DNA hydroxymethylation may play an essential role in the pathology of AR. In line with our results, Somineni et al. (39) revealed that TET1 expression and global 5hmC levels were higher in both nasal airway epithelial cells and PBMCs of asthma patients. Global DNA methylation and hydroxymethylation levels in the lung were also increased in a murine model of HDM-induced airway hyperresponsiveness (40). AR and atopic asthma, which are considered as united airway disease, have been recognized to share similar underlying pathogenesis and immunologic mechanisms (41, 42) including, perhaps, epigenetic regulation.

AR is distinguished by a lack of immunological tolerance toward the specific allergen. We induced moDCs from HDM-sensitive AR patients and found that the major HDM allergen, Der p 1, significantly decreased TET1 expression in atopic moDCs. Our study suggests the hypermethylated DNA status during maturation of DCs is due not only to the increased DNA methyltransferase 3 beta (DNMT3B) (29) but also the inhibited DNA demethylation enzyme TET1. This study deepens our understanding of AR by delineating the role of TET enzymes in mediating allergen challenge effect. We knocked down the predominant TET (TET1) in moDCs and found that TET1-deficient atopic DCs is more susceptible to be activated than non-atopic DCs. Relevantly, Zhang et al. (24) established that several inflammatory mediators, including IL-6, were significantly upregulated in TET-silenced DCs. Our present study shows TET1-deficient moDCs suppressed conversion of naïve T cells into aTregs, the activated subset of regulatory T cells and are essential in maintaining immune tolerance and immune homeostasis, especially in those with AR (30). Recently, Burleson et al. (43) established that HDM-challenged TET1-deficient mice have impaired interferon pathway, increased airway hyperresponsiveness, and eosinophilic infiltration in the lung. Together, this evidence suggests that the loss of TET1 induces the maturation of DCs and mediates the development of allergic airway inflammation.

Superficially, our observation of higher baseline TET1 expression in AR group while also showing TET1 inhibition that induces DC activation in AR seem to be contradictory. However, the observation was based on a cross-sectional study. One should not assume that the higher TET1 expression in AR would be reflected similarly in the context of acute allergen challenge. Even though the levels of TET1, TET3, and 5hmC are higher in the PBMCs of AR patients, the difference between 5mC and 5hmC in the PBMCs of AR (shown in Figure S1) favors 5mC, inferring that the balance of 5mC/5hmC polarizes to DNA hypermethylation in AR patients. Accordingly, we have two hypotheses to resolve these observations. Firstly, the higher TET1 in AR group might be driven by underlying genetics rather than by environmental exposures. Consistent with this hypothesis, Somineni et al. (39) showed that asthmatics are typically hypomethylated at CpG cg23602092 in the TET1 promoter, which usually infers higher expression of TET1. A second possibility to explain the baseline status we observed regarding the higher expression of TETs in AR patients is a chronic homeostatic regulation, opposite the acute response we demonstrated experimentally, drives higher levels of TET. However, these hypotheses regarding differences between a stable vs. antigen-challenge environment remain the subject of future investigation.

This study has several limitations. Firstly, since the restriction of obtaining the nasal mucous tissues from human volunteers, and the infiltration level of DCs in nasal mucosa is low, it is impractical to quantify the expression of TET in local infiltrated DCs in AR patients. Therefore, we sampled the PBMCs to explore TET expression in DCs by flow cytometry. Secondly, the roles of TET in other cell subtypes need to be further determined. Moreover, this study was based on clinical cross-sectional observation and *in vitro* experiments. In the future, we will establish *in vivo* studies to investigate the environmental factors and allergen challenge effects on TET expression and its epigenetic regulation in the immune response.

In this study, TET1 and DNA hydroxymethylation were shown to come into play in the immune activation of AR. Revealing the role of TET enzymes and their specific target genes in particular cells or tissues will help researchers to clarify the molecular mechanism in the development of diseases. Furthermore, knowing the target sequences of TET enzymes, researchers can use epigenome editing tools, such as CRISPR/dCas9 system, to selectively regulate gene expression and biological activities (44).

## Conclusions

This study suggests that DCs from AR patients express higher TET1 and are susceptible to be activated by TET1 decrease, which can be triggered by allergen challenge. TET-mediated DNA demethylation is involved in the pathogenesis of AR, highlighting the epigenetic regulation in the development of allergic airway inflammation. These findings encourage us to explore the potential environmental elements that may mediate the epigenetic regulation in AR and targeting DNA demethylation regulation could help find more effective methods for AR prevention and treatment.

## Ethics Statement

This study was carried out in accordance with the recommendations of, and the protocol was approved by, the Ethics Committee of The First Affiliated Hospital of Sun Yat-sen University, Guangzhou, China (Approval No. 2016197). All subjects gave written informed consent in accordance with the Declaration of Helsinki.

## Author Contributions

HL, WS, and WW designed the study. RM, DC, and YWen collected and processed the clinical specimens. HL, TL, and YWei conducted the experiments. HL and HZ prepared the figures and tables. HL and WS drafted the manuscript. WW and CC revised the manuscript. All authors read and approved the final manuscript.

### Conflict of Interest

The authors declare that the research was conducted in the absence of any commercial or financial relationships that could be construed as a potential conflict of interest.
