# Supplementary material for: Ten-Eleven Translocation (TET) Enzymes Modulate the Activation of Dendritic Cells in Allergic Rhinitis
Source: Front Immunol. 2019 Sep 26;10:2271. doi: 10.3389/fimmu.2019.02271 (PMC6775386; doi:10.3389/fimmu.2019.02271)
Supplement: Supplementary file 1 [file Table_1.DOCX]

Supplementary Material

Ten-eleven translocation (TET) enzymes modulate the activation of dendritic cells in allergic rhinitis

**Hang Li^1,2^*, Tong Lu^1,2^*****, Wei Sun^1,2^*, Renqiang Ma^1,2^, Hua Zhong^1,2^, Yi Wei^1,2^, Dehua Chen^1,2^, Yihui Wen^1,2^, Chris Carlsten^3‡^, Weiping Wen^1,2‡^**

^1^ Department of Otorhinolaryngology Head and Neck Surgery, the First Affiliated Hospital of Sun Yat-sen University, Guangzhou, China;

^2^ Guangzhou Key Laboratory of Otorhinolaryngology Head and Neck Surgery, the First Affiliated Hospital of Sun Yat-sen University, Guangzhou, China;

^3^ Air Pollution Exposure Laboratory, Division of Respiratory Medicine, Department and Faculty of Medicine, University of British Columbia, Vancouver, Canada.

*These authors contributed equally to this work as first authors.

‡These authors contributed equally to this work as corresponding authors

**Correspondence:**

Weiping Wen

wenwp@mail.sysu.edu.cn

Chris Carlsten

carlsten@mail.ubc.ca

# Supplementary Methods

**Quantitative real-time PCR (qPCR)**

The mRNA expression levels of TET1, TET2, and TET3 were evaluated using qPCR. PBMCs were isolated, from peripheral blood of the subjects, by Ficoll-Hypaque density gradient centrifugation with Lymphocyte Separation Medium (MP Biomedicals, Santa Ana, USA). RNA purification from PBMCs was performed with the RNAiso Plus reagent (TAKARA Bio, USA) according to the manufacturer’s instructions. cDNA was obtained by using reverse transcription kit (TAKARA Bio, USA). The mRNA expression was tested using an ABI PRISM 7500 Detection System (Applied Biosystems, CA) and SYBR Premix (Roche). The primer sequences (5’-3’) are listed in supplementary material Table S2. PCR reactions were performed in a total volume of 20 μL containing 10 μL SYBR Green Master Mix, 1μL of 5 mmol/L primers, and 25 ng synthesized cDNA. Reactions ran according to the manufacturer’s instructions. mRNA levels were normalized to GAPDH. The relative mRNA levels of the target genes were analyzed by using the 2^-ΔΔCt^ method. Experiments were performed in triplicate for each data point.

**Quantification of 5mC and 5hmC**

Genomic DNA was isolated from PBMCs by using E.Z.N.A. Genomic DNA isolation kit (Omega Bio-Tek). Global 5mC and 5hmC content in the PBMCs were quantified with 5mC DNA ELISA Kit and Quest 5hmC DNA ELISA kit (ZYMO, USA). According to the manufacturer’s instructions of 5mC DNA ELISA kit, 100ng of each DNA was added to a PCR tube and diluted with 5mC Coating buffer to 100 μL final volume. DNA was denatured at 98℃ for 5 mins and transferred immediately to ice for 10 mins. Entire volume denatured DNA was added to the wells of the plate, cover with foil, and incubated at 37℃ for 1 hour. The buffer was discarded, and the wells were washed 3 times with 200 μL of 5mC ELISA buffer. 200 μL of 5mC ELISA buffer was added to each well and incubated at 37℃ for 30 mins. The buffer was discarded, and each well was incubated with the anti-5-Methylcytosine antibody (1:2000) and secondary antibody (1:1000) at 37℃ for 1 hour. After washed 3 times with 5mC ELISA buffer, each well was added with 100 μL of HRP Developer and incubated at room temperature (RT) for 1 hour. Absorbance was measured at 405 nm. 5hmC content was determined following the kit’s instructions.

**Western blot analysis**

Cells were lysed using RIPA (Millipore, Billerica, USA) containing protease inhibitor cocktail (Sigma-Aldrich, St Louis, USA). Protein concentration was determined by BCA Protein Assay Kit (Beyotime Biotechnology, Nantong, Jiangsu, China). 30μg protein of each sample was separated using SDS-PAGE (8% gel) and then transferred onto PVDF membrane (Millipore, Billerica, USA). The membrane was blocked with 5% BSA at RT for 1 h and incubated with primary antibody at 4℃ overnight. The membrane was washed with TBST and incubated with HRP-conjugated secondary antibody at RT for 1 h. After washed with TBST, the membrane was visualized by using an ECL kit (Millipore, Billerica, USA). The results were quantified using densitometry (Image J). TET1 (1:1000, ab191698, Abcam, Cambridge, MA, USA), TET2 (1:1000, ab94580, Abcam), TET3 (1:1000, ab139311, Abcam) and HRP-conjugated GAPDH monoclonal antibody (1:10000, proteintech) were used in this experiment.

# Supplementary Figures and Tables

## Supplementary Figures


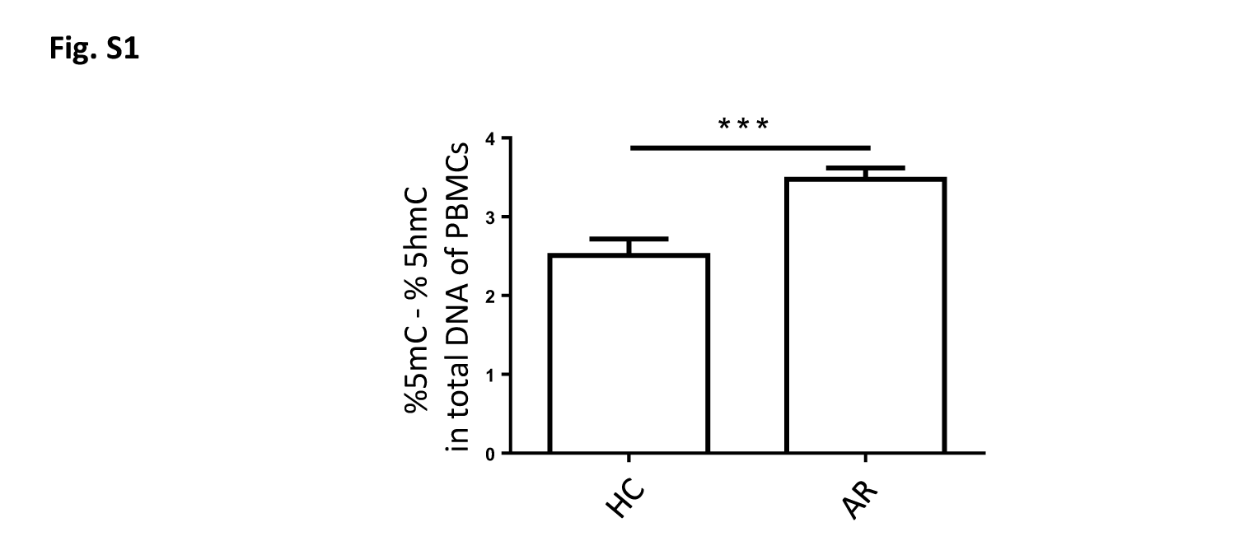


**Figure S1. Difference between 5mC and 5hmC in the PBMCs of allergic rhinitis (AR) patients comparing with healthy controls.** The difference between the levels of global 5mC and 5hmC in the PBMCs of healthy controls and AR patients were evaluated. Values were presented as means ± SEMs. Mann-Whitney tests were conducted. *** *p* <0.001.


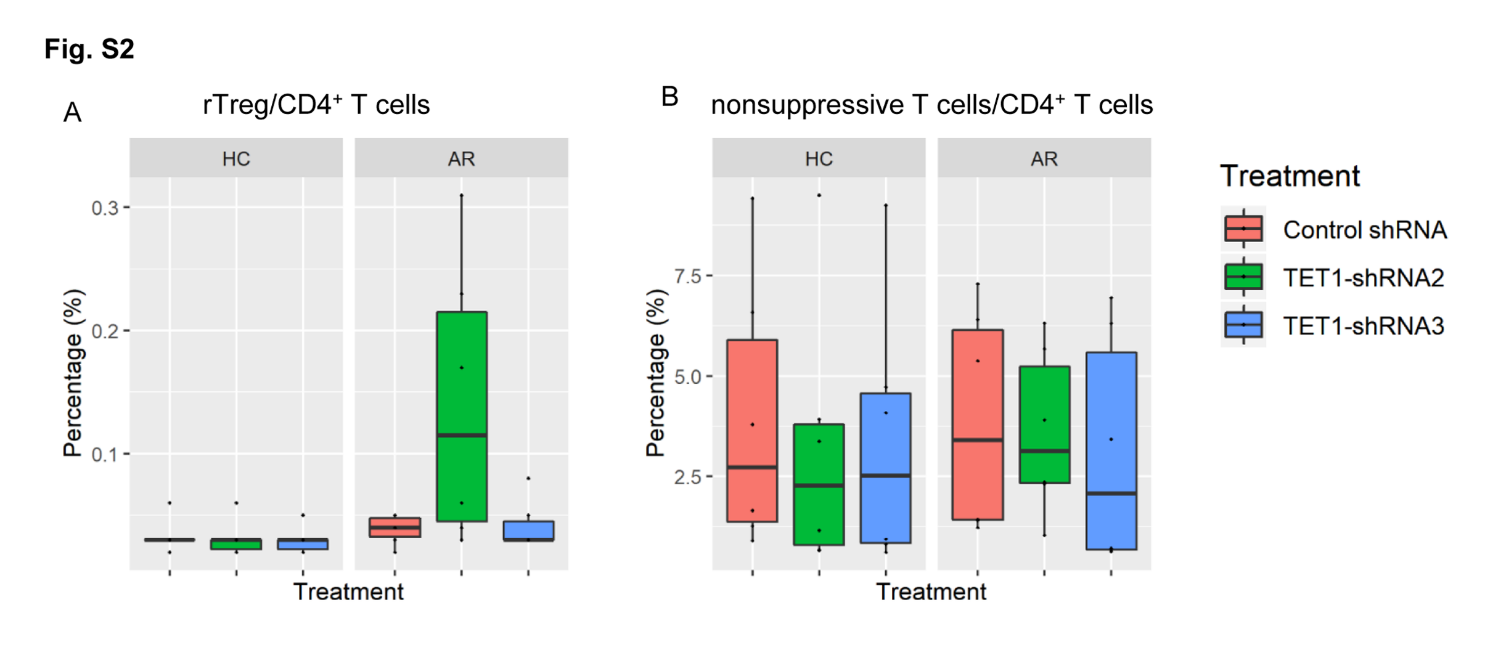


**Figure S2. TET1-inhibited moDCs had no significant effect on the differentiation of CD4^+^CD45RA^+^FoxP3^lo^ resting Treg cells and CD4^+^CD45RA^-^FoxP3^lo^ nonsuppressive T cells.** Immature non-atopic and atopic moDCs (day 5) were transfected by control-shRNA, TET1-shRNA2, or TET1-shRNA3 adenovirus. On day 7, moDCs were co-cultured with autologous CD4^+^ T cells at a ratio of 1:10 (1×10^4^: 1×10^5^) for another 72 hrs. The proportion of CD4^+^CD45RA^+^FoxP3^lo^ resting Treg cells (rTregs) (A), and CD4^+^CD45RA^-^FoxP3^lo^ nonsuppressive T cells (B) in CD4^+^ T cells were evaluated. Statistical analysis were conducted with Friedman test and Dunn's multiple comparisons tests. (n=6 HC and 6 AR)

**Figure S3. Original WB image of Figure 3B.**

**
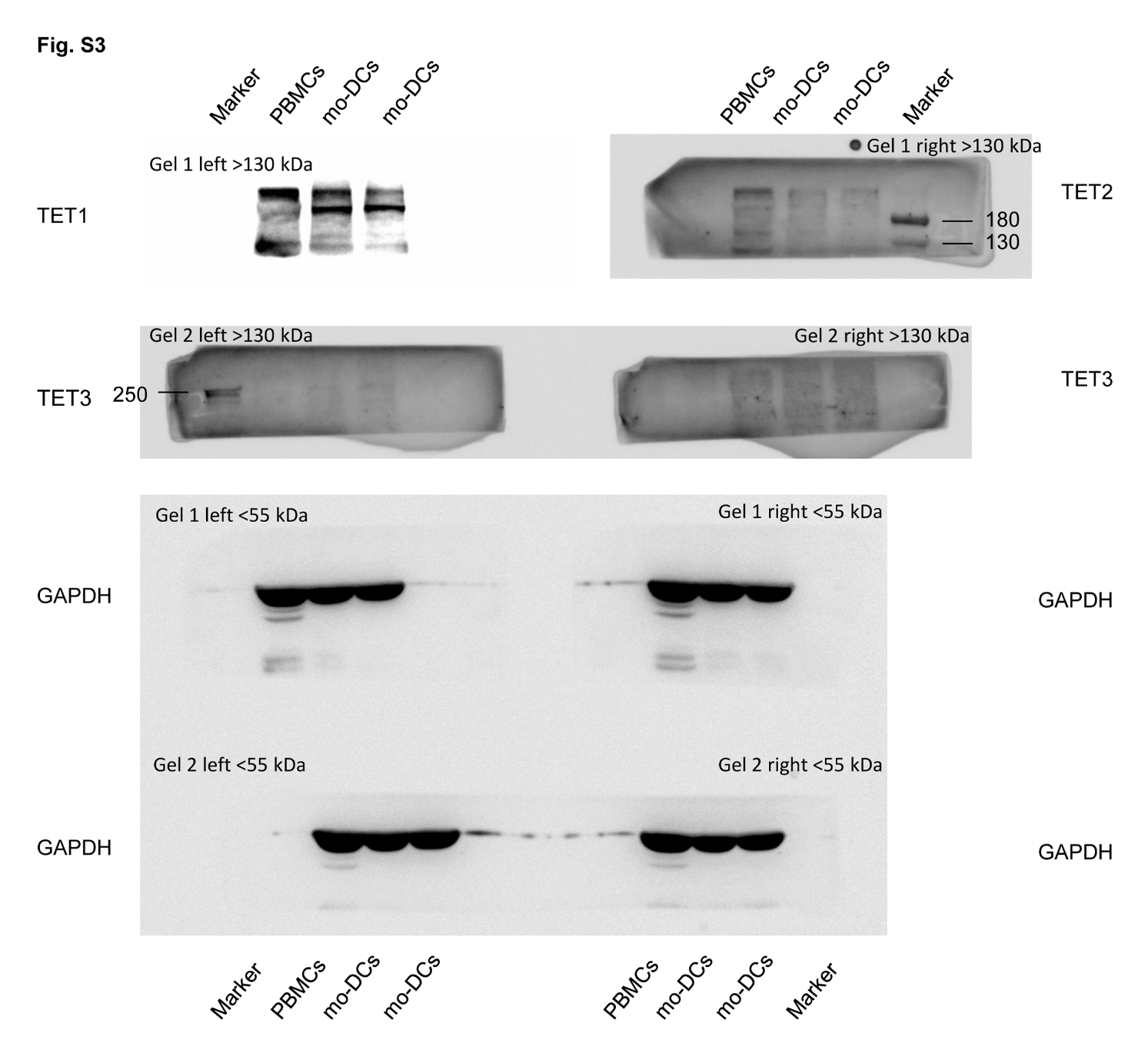
**

**Figure S4. Original WB image of Figure 3D**

**
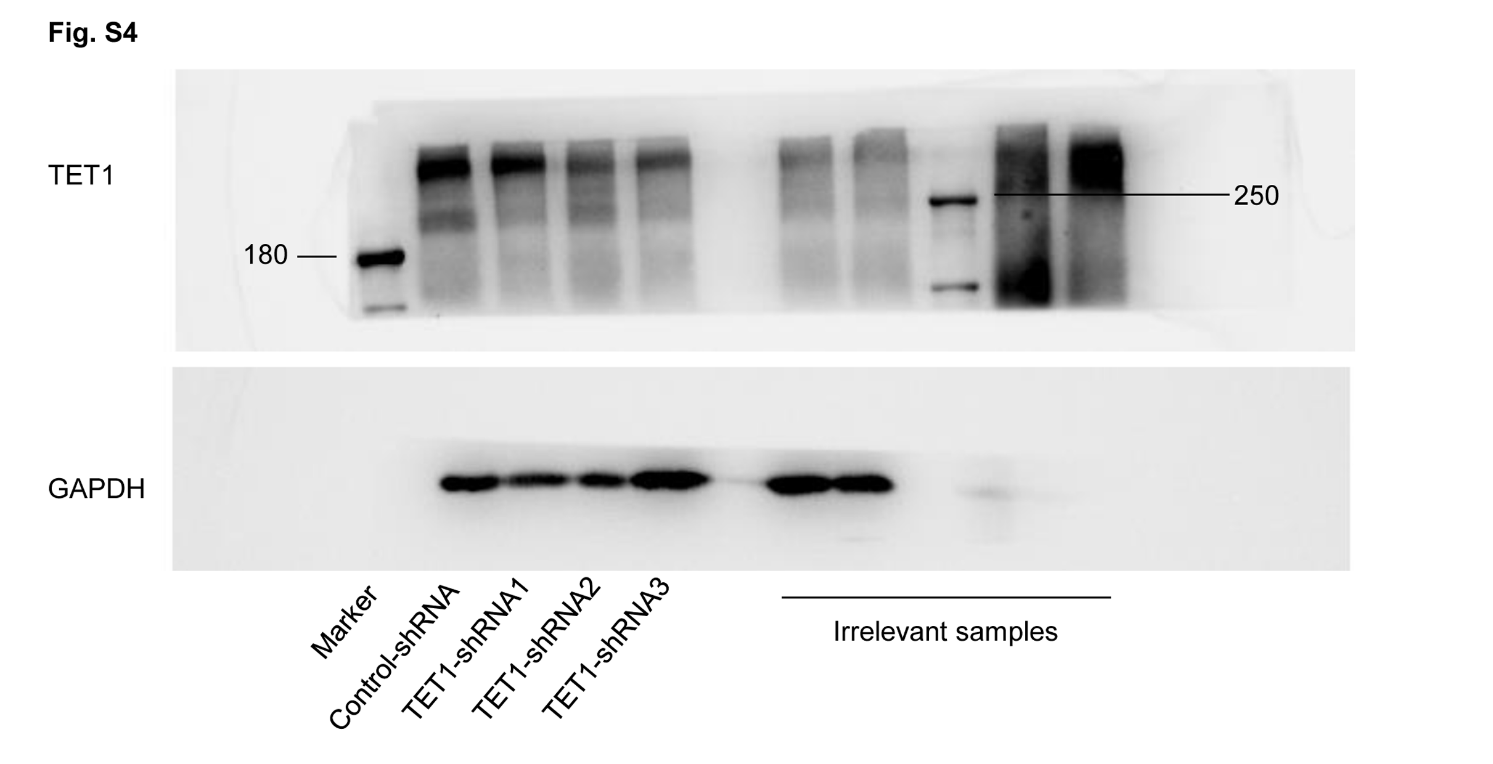
**

## Supplementary Tables

### Table S1. Summary of clinical sample size used in each experiment

|  | Healthy volunteers  (n=38) | Allergic rhinitis  (n=94) |
| --- | --- | --- |
| qPCR | 23 | 69 |
| DNA 5mC and 5hmC quantification* | 13 | 32 |
| Flow cytometry | 15 | 25 |

* Subjects tested with DNA 5mC and 5hmC quantification were included in qPCR.

### Table S2. Primer sequence for qPCR

| Gene name | Primer sequence |
| --- | --- |
| Human-TET1- Forward primer | CTGGCTCAAACGAGGTCCAT |
| Human-TET1- Reverse primer | TGCCATCACGTTAGCACACT |
| Human-TET2- Forward primer | AGGCTAGGCTGCTTTCGTAG |
| Human-TET2- Reverse primer | GAATGTTTGCCAGCCTCGTT |
| Human-TET3- Forward primer | AGTGGCTTCTTGGAGTCACCTC |
| Human-TET3- Reverse primer | GGATGGCTTTCCCCTTCTCTCC |
| Human-GAPDH-Forward primer | GAGTCAACGGATTTGGTCGT |
| Human-GAPDH-Reverse primer | TTGATTTTGGAGGGATCTCG |
